# The impact of community-wide, mass drug administration on aggregation of soil-transmitted helminth infection in human host populations

**DOI:** 10.1186/s13071-020-04149-4

**Published:** 2020-06-08

**Authors:** Marleen Werkman, James E. Wright, James E. Truscott, William E. Oswald, Katherine E. Halliday, Marina Papaiakovou, Sam H. Farrell, Rachel L. Pullan, Roy M. Anderson

**Affiliations:** 1 London Centre for Neglected Tropical Disease Research (LCNTDR), Department of Infectious Disease Epidemiology, St. Mary’s Campus, Imperial College London, London, UK; 2 The DeWorm3 Project, The Natural History Museum of London, London, UK; 3 MRC Centre for Global Infectious Disease Analysis, School of Public Health, Imperial College London, London, UK; 4 Present Address: Centre for Global Child Health, Hospital for Sick Children, Toronto, Canada; 5 Faculty of Infectious and Tropical Diseases, London School of Hygiene & Tropical Medicine, London, UK; 6 Department of Life Sciences, Natural History Museum, London, UK

**Keywords:** Soil-transmitted helminths, Aggregation, Stochastic simulations

## Abstract

**Background:**

Soil-transmitted helminths (STH) are intestinal parasites estimated to infect over 1.5 billion people. Current treatment programmes are aimed at morbidity control through school-based deworming programmes (targeting school-aged children, SAC) and treating women of reproductive age (WRA), as these two groups are believed to record the highest morbidity. More recently, however, the potential for interrupting transmission by treating entire communities has been receiving greater emphasis and the feasibility of such programmes are now under investigation in randomised clinical trials through the Bill & Melinda Gates Foundation funded DeWorm3 studies. Helminth parasites are known to be highly aggregated within human populations, with a small minority of individuals harbouring most worms. Empirical evidence from the TUMIKIA project in Kenya suggests that aggregation may increase significantly after anthelminthic treatment.

**Methods:**

A stochastic, age-structured, individual-based simulation model of parasite transmission is employed to better understand the factors that might induce this pattern. A simple probabilistic model based on compounded negative binomial distributions caused by age-dependencies in both treatment coverage and exposure to infection is also employed to further this understanding.

**Results:**

Both approaches confirm helminth aggregation is likely to increase post-mass drug administration as measured by a decrease in the value of the negative binomial aggregation parameter, *k*. Simple analytical models of distribution compounding describe the observed patterns well.

**Conclusions:**

The helminth aggregation that was observed in the field was replicated with our stochastic individual-based model. Further work is required to generalise the probabilistic model to take account of the respective sensitivities of different diagnostics on the presence or absence of infection.
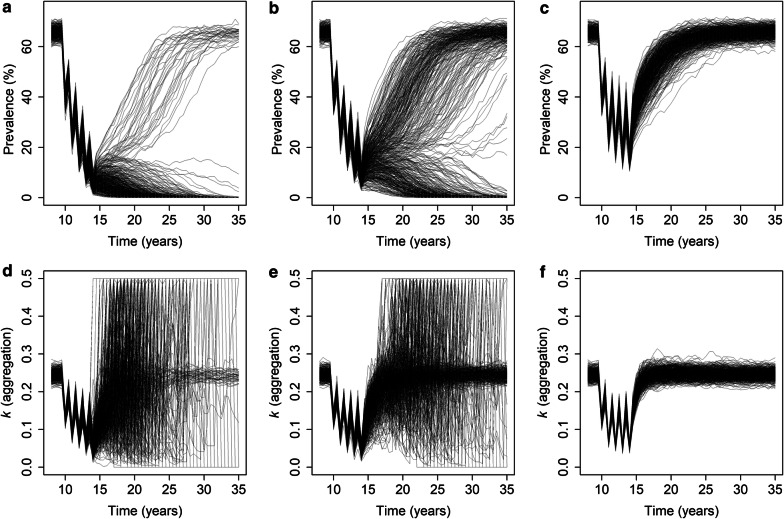

## Background

The soil-transmitted helminths (STH) are a group of intestinal parasites comprising *Ascaris lumbricoides* (roundworm), *Trichuris trichiura* (whipworm), and *Necator americanus* and *Ancylostoma duodenale* (the hookworms). STH are thought to induce the largest morbidity burden of all neglected tropical diseases (NTDs) [1], with chronic medium- to high-intensity infections associated with growth retardation and intellectual impairment in children, and anaemia. There are an estimated 1.5 billion individuals infected with at least one intestinal nematode globally, cumulatively resulting in over five million disability-adjusted life years (DALYs) [2]. The greatest burden of STH infection falls upon those of lowest socioeconomic status in Southeast Asia and sub-Saharan Africa [3]. The most common anthelmintic drugs in use against STH are albendazole and mebendazole. Both are known to have few side effects [4, 5] generally high efficacy [6], and are currently donated for global use in de-worming programmes by GlaxoSmithKline and Johnson & Johnson, respectively.

The World Health Organisation (WHO) STH treatment guidelines currently target morbidity control, primarily through repeated, school-based de-worming programmes and aim to treat women of reproductive age (WRA) [7]. However, finding and treating WRA can be challenging [8]. More recently, however, a debate has started around the possibility of eliminating transmission, in addition to morbidity control, by expanding treatment across a broad class of age groups including, crucially, the adult population. This shift in focus was in part triggered by the London Declaration in 2012 which brought attention to the control of NTD morbidity by 2020, and possible eradication of transmission of certain infections in some settings [9].

Previously published results have shown that interruption of transmission is unlikely to be achieved when only treating school-aged children (SAC) and WRA, since the untreated adult population will continue to contribute to the reservoir of infectious material thus causing reinfection amongst SAC [10–13]. This is especially the case for hookworm, where the majority of worms are harboured by adults [13], but it also applies to a lesser extent for the other prevalent STH species *A. lumbricoides* and *T. trichiura* [10]. It is important to note that even though WRA suffer more from morbidity than the adult male population, untreated adult males may harbour significant worm populations. Therefore, just treating SAC and WRA is unlikely to result in the interruption of transmission.

Successfully interrupting transmission would stop the need for long-term MDA in areas of endemic infection. Faced with the prospect of a never-ending dependence on MDA, countries are starting to consider expanding de-worming efforts beyond current school-based approaches [14–17]. As a result, greater emphasis has been placed upon implementing community-wide mass drug administration (MDA) de-worming programmes. The impact of successive rounds of MDA on STH prevalence is the primary focus of the Bill & Melinda Gates Foundation funded DeWorm3 project in India, Malawi and Benin [18]. This project is investigating whether a community-wide MDA-only approach to STH treatment, would be sufficient to reach a prevalence threshold below which helminth parasite populations cannot sustain transmission, thus leading to transmission elimination [19–21]. All STH species reproduce sexually within a human host to produce fertile eggs. When the mean worm burden in a population falls to very low levels, the likelihood of both a male and female worm residing within a single human host becomes very small. A “breakpoint” is reached when the worm population can no longer sustain reproduction, resulting in the interruption of transmission even in the absence of further treatment [20, 21].

Helminth parasites are highly aggregated within human populations, with a small minority of individuals harbouring the majority of worms [22–27]. This “clumping” increases the likelihood that parasites successfully locate members of the opposite sex within a human host. Hence, the breakpoint in infection is much lower when parasites are aggregated as compared to randomly distributed throughout the host population. The aggregation parameter of the negative binomial distribution, *k*, inversely measures the extent of “clumping” or worm aggregation present in the human host population. The smaller the value of *k*, the greater the extent of aggregation [13]. As MDA coverage intensifies in endemic regions, it becomes more important to understand how the increased treatment pressure impacts the pattern of parasite aggregation, and whether any changes can help provide information to assist in improving the design of community-based MDA programmes. In this study, we investigate the impact of MDA on worm aggregation *via* observed patterns in communities experiencing high MDA coverage; through the predictions of probability models of the impact of MDA; and through individual-based stochastic simulations of parasite transmission and MDA impact under different patterns of individual compliance to multiple rounds of treatment.

Previous work has suggested that the probability with which repeated rounds of MDA will successfully result in transmission elimination is highly dependent on both treatment coverage, i.e. the proportion of individuals taking medication at any one round of treatment [28], and treatment compliance, i.e. the proportion of MDA rounds that each individual takes treatment [29, 30]. Past research has shown that the impact of a community-based MDA programme is greatly influenced by the specific pattern of non-compliance [30]. At one end of the spectrum it could be random, where all individuals have an equal chance of being treated at each round. At the other extreme is systematic non-compliance whereby the same individuals do not take treatment at any round of MDA. An intermediary pattern is semi-systematic in which each individual is assigned a lifelong probability of taking treatment at each round [30, 31]. Each of these non-compliance patterns may affect observed parasite aggregation patterns. Therefore, the analyses reported in this paper also examine the impact of different MDA compliance scenarios on the aggregation of parasites within the human host.

The overall aim of this study is to determine whether repeated, community-wide anthelminthic treatment has an impact on the degree of parasite aggregation and, if so, whether this impact can be predicted as a function of MDA coverage and compliance. We also discuss the policy implications of increased parasite aggregation post-MDA, as the prevalence of infection declines to low levels.

## Methods

**Fig. 1:**
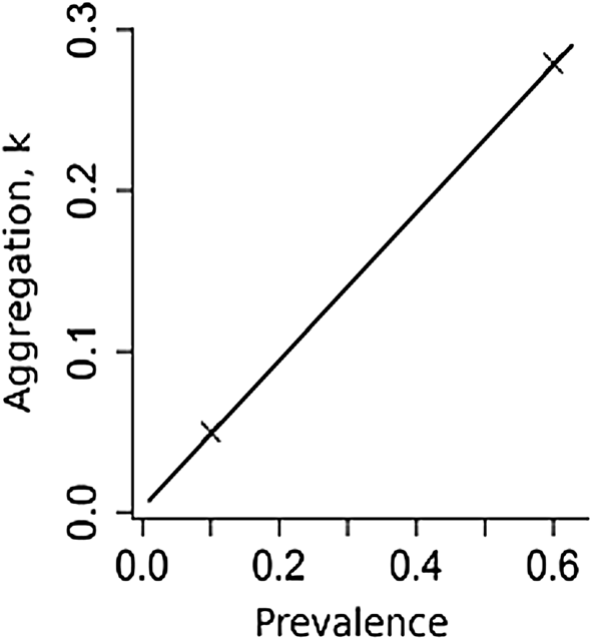
Observed relationship between measured prevalence and aggregation, *k*, from TUMIKIA project. Originally published in Truscott et al. [34]. The crosses in represent the prevalence values of 10% and 60%. In the TUMIKIA study, the lowest *k*-values were found in low prevalence clusters (< 10%) and the highest *k*-values were found in clusters with a prevalence of approximately 60%

Empirical data derived from a large-scale field epidemiological study provided the stimulus for this research on parasite aggregation. The TUMIKIA project was a cluster-randomised, controlled trial conducted in Kwale County, Kenya, from March 2015 to May 2017. The Kwale County has received MDA to control lymphatic filariasis, which is also effective for the control of STH. Annual school-based MDA has been provided to this county three years before the start of the trial. The trial compared the impact on STH infection of annual and biannual MDA, *versus* annual mass anthelmintic treatment of children (2–14 years-old) [32, 33]. Recently, Truscott et al. [34] investigated the heterogeneity in transmission parameters from the TUMIKIA trial. Data obtained from the TUMIKIA study provided evidence of changes in the negative binomial aggregation parameter *k*, under intensive MDA. Clusters with a low prevalence were found to have a higher degree of parasite aggregation [34] (Fig. 1). In this study, we further investigate the changes in parasite aggregation. Since observations from the TUMIKIA study related to *N. americanus*, this will be the parasite of reference in this paper. However, results relating to *A. lumbricoides* will also be summarised, with further detail and figures provided in Additional file 1: Figures S1–S5.

We employ an event-driven, individual-based stochastic model of helminth transmission [30], building on a model framework originally described by Anderson & May [35] and Anderson & Medley [36]. The model includes age-dependent infection rates, density-dependent worm fecundity, parasite sexual reproduction which is assumed to be polygamous, and a negative binomial distribution of parasites per person [37]. The latter is created by assigning an exposure risk factor to individuals drawn from a gamma distribution for a specific age-grouping (the compounding of Poisson distributions where the means are drawn from a gamma distribution [38]). It is the dynamics of sexual reproduction for dioecious species which results in the occurrence of three possible equilibria in the deterministic dynamical system; a stable endemic state, and parasite extinction, separated by an unstable “breakpoint” in transmission below which the attractor is extinction [27]. We denote this as a prevalence threshold below which the parasite population cannot sustain transmission. Once this state is reached, there is no need for further MDA assuming an absence of infective individuals migrating to the area who could re-populate the reservoir of infection. In a stochastic world, the deterministic concept of a breakpoint needs expansion to a small area around the deterministic breakpoint where the system, due to chance events, may move to either stable equilibrium. The individual-based, stochastic modelling of events *via* numerical studies allows for the explicit inclusion of heterogeneity between individuals, such as in treatment compliance and exposure to infection, as well as probabilistic forecasts of the likely impact of different MDA programmes in a variety of transmission settings (e.g. low, medium and high transmission settings as defined by the value of the basic reproductive number, R_0_).

The parameter values employed in the individual-based, stochastic simulation model for *N. americanus* and *A. lumbricoides* are presented in Table 1. Each stochastic model-based simulation comprised 1000 villages, each containing 1000 individuals. All villages underwent five rounds of annual, community-wide MDA, with coverage levels for *N. americanus* set at 85% for both pre-school-aged children (pre-SAC) and SAC, and 75% for adults, whilst for *A. lumbricoides* coverage was 75% for pre-SAC and SAC, and 30% for adults. These coverage levels were selected to ensure that a proportion of simulations achieved elimination and a proportion did not, such that the validity of the predictions of the simple analytical model could be compared with those of the more complex individual stochastic model in both scenarios. Simulations were run for 10 years pre-treatment and 30 years post-treatment to ensure STH prevalence had reached equilibrium both before and after anthelmintic treatment was provided.

**Table 1:** Parameters used for the individual-based, stochastic simulation models

Model parameter description	*A. lumbricoides*	*N. americanus*
Transmission rate, R_0_	2.12	2.2
Aggregation of worms within human hosts, *k*	0.15	0.35 [25]
Relative exposure to infection and contribution to infective reservoir (assuming no differences between males and females)	0–2 years-old: 0.22	0–2 years-old: 0.03
	2–5 years-old: 1.88	2–5 years-old: 0.09
	5–15 years-old: 1	5–15 years-old: 1
	15+ years-old: 0.53	15+ years-old: 2.5 [49]
Average worm life expectancy (years) (assuming an exponential distribution)	1 [50]	2 [51]
Female worm fecundity, γ	0.07 [37]	0.02 (assuming exponential saturation) [52]
Infectious reservoir decay rate (mean)	2 months [35]	12 days [51]
Drug efficacy	0.99 [40]	0.948 [40]

At each time point, the effective negative binomial aggregation parameter is calculated by finding the maximum likelihood estimate $\hat{k}$ from the distribution of worm burdens amongst hosts [39]. These estimates are subsequently presented as line graphs showing how worm aggregation changes over time. STH prevalence is also calculated for each village at each time points.

The impact of treatment on parasite aggregation can also be expressed in a simplified probability model from which some key analytical results can be derived. In a homogenous population, worm burdens among hosts are precisely negative binomial, with a fixed *k* value within our simulation. However, once heterogeneities are introduced, such as age structure and differing uptake of MDA within the population, this is no longer the case. Worm burden distributions after MDA or across wide age ranges with differing exposure intensities can be described by a compound distribution, in which the underlying negative binomial distribution is compounded by the probability distribution associated with the given source of heterogeneity. Using this approach, we can approximate the impact of these mechanisms on aggregation by calculating the changes to both the mean and variance of the worm burden among hosts that they influence. For a negative binomial distribution, the relationship between the aggregation parameter *k*, the mean µ and the variance σ^2^ is given by

$$k = \frac{{\mu^{2} }}{{\sigma^{2} - \mu }}.$$

Under the effect of heterogeneity, the new values of mean worm burden and variance can be used to calculate an effective aggregation parameter, *k*′, under the assumption that the worm distribution is still approximately negative binomial.

In the case of MDA with imperfect coverage, a round of treatment splits the target population into two sub-populations: a treated group with reduced worm burden and an untreated group whose worm burden is assumed to be unchanged. Using the technique described by Anderson & May [27], for a round of MDA, with coverage *c* and efficacy *e*_*f*_, applied to a homogeneous host sub-population with mean worm burden *m* and aggregation *k*, the approximate aggregation post-treatment is

1 $$k^{\prime} \simeq k\frac{{(1 - e_{f} c)^{2} }}{{\left( {k + 1} \right)e_{f}^{2} c\left( {1 - c} \right) + (1 - e_{f} c)^{2} }}.$$

By extending this approach, we can also incorporate the heterogeneity induced by age-dependent exposure to infection. For further information, please see Additional file 2: Text S1.

If we consider the impact of treatment across a range of ages where there is a significant difference in exposure to infection, a further level of variability is introduced into the calculation. If we construct *N* age categories, the probability of sampling (randomly) from the *i*^th^ is

$$p\left( i \right) = \frac{{\# {\text{people in }}i^{th} {\text{age group}}}}{\text{Total population}}$$

where the value of m_0_ and *c* are functions of the age-group *i*. The new variance of worm burden now becomes

$$Var\left[ w \right]' = E_{i} E_{m} [Var_{w} \left[ {w\left( m \right)} \right]\left] {\left] { + E_{i} } \right[Var_{m} } \right[E_{w} \left[ {w\left( m \right)} \right]\left] {\left] { + Var_{i} } \right[E_{m} } \right[E_{w} \left[ {w\left( m \right)} \right]]]$$

This can be expressed in terms of the previous simpler expression for the variance

$$Var\left[ w \right]' = E_{i} \left[ {Var\left[ w \right]\left( {m_{0} \left( i \right),c\left( i \right)} \right)} \right] + Var_{i} \left[ {\bar{m}\left( {m_{0} \left( i \right),c\left( i \right)} \right)} \right]$$

Since coverage levels and mean worm burden are both functions of age group, this expression cannot be evaluated in closed form.

The expected *k* values, mean worm burdens and variance in worm counts immediately post-treatment are calculated from the pre-treatment data obtained from the simulations. These expected post-treatment values are then compared to those observed from the simulations to determine the validity of the simple analytical predictions. Scatterplots are used to visually portray the comparisons.

## Results

### Predictions based on the stochastic simulations

**Fig. 2:**
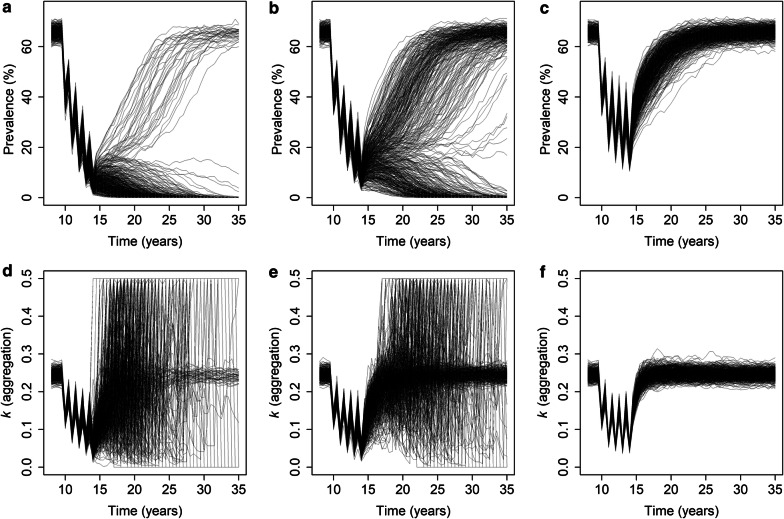
Prevalence of *Necantor americanus* over time (**a**–**c**) and aggregation (parameter *k*) over time (**d**–**f**), in this plot we show 500 out of 1000 simulated villages. **a** and **d**: Random non-compliance; **b** and **e**: Semi-systematic non-compliance; **c** and **f**: Fully-systematic non-compliance

At equilibrium, *N. americanus* prevalence was approximately 65% in all villages for all compliance scenarios. After cessation of treatment, village prevalence either recovered to pre-treatment equilibrium levels or decreased to zero, depending on whether the breakpoint in transmission was crossed. The proportion of villages achieving *N. americanus* elimination was 89.4% under random non-compliance, 41.7% for semi-systematic compliance and 0% for fully-systematic compliance (Fig. 2a–c). For *A. lumbricoides* the equivalent proportions were 75.4%, 65.6% and 42.8%, respectively (Additional file 1: Figure S1a–c). These results clearly illustrate the importance of individual compliance as a key determinant of MDA impact. This effect is not widely appreciated by those who track MDA coverage as reported on a country to country basis to WHO. In the WHO compilations of year by year progress in MDA, coverage is documented but not compliance.

Under random non-compliance, *k* decreased sharply (reflecting increased parasite aggregation) to between 0.102 and 0.160 directly following the first round of treatment. Similar patterns were also observed in both the fully-systematic and semi-systematic non-compliance scenarios (range: 0.100–0.160 and 0.100–0.151, respectively). In all scenarios, *k* became successively smaller after each round of treatment, with the recovery in *k* immediately post-treatment consistently lower than at each previous round. Furthermore, the range in *k* values recorded in the stochastic simulations increased slightly after each successive round of treatment. In villages where elimination of transmission was successful (the “breakpoint” in transmission was crossed), the prevalence declined to zero and *k* concomitantly decayed to a value of zero. On average, this took 12 years post-cessation of treatment for random non-compliance (range: 3.0–21.0 years) and 14.5 years for semi-systematic (range: 6.5–21.0 years). No simulations in the fully-systematic non-compliance scenario resulted in elimination, hence no *k* values reached zero.

For *A. lumbricoides*, the duration of time for *k* to reach zero post-treatment was 6.5 years for random non-compliance (range: 0.5–20.0 years), 7.0 years for semi-systematic compliance (range: 1.5–19.5 years), and 8 years for fully-systematic non-compliance (range: 3–21.0 years). In those villages which failed to reach elimination and suffered bounce-back in infection to pre-treatment equilibrium levels, *k* was observed to also return to its pre-treatment value. On average, this bounce-back took approximately 6 years post-cessation of treatment for both *N. americanus* (Fig. 2d–f) and *A. lumbricoides* (Additional file 1: Figure S1d–f). No significant difference was observed between different compliance patterns with regards to the duration of time required for *k* to return to pre-treatment equilibrium levels.

**Fig. 3:**
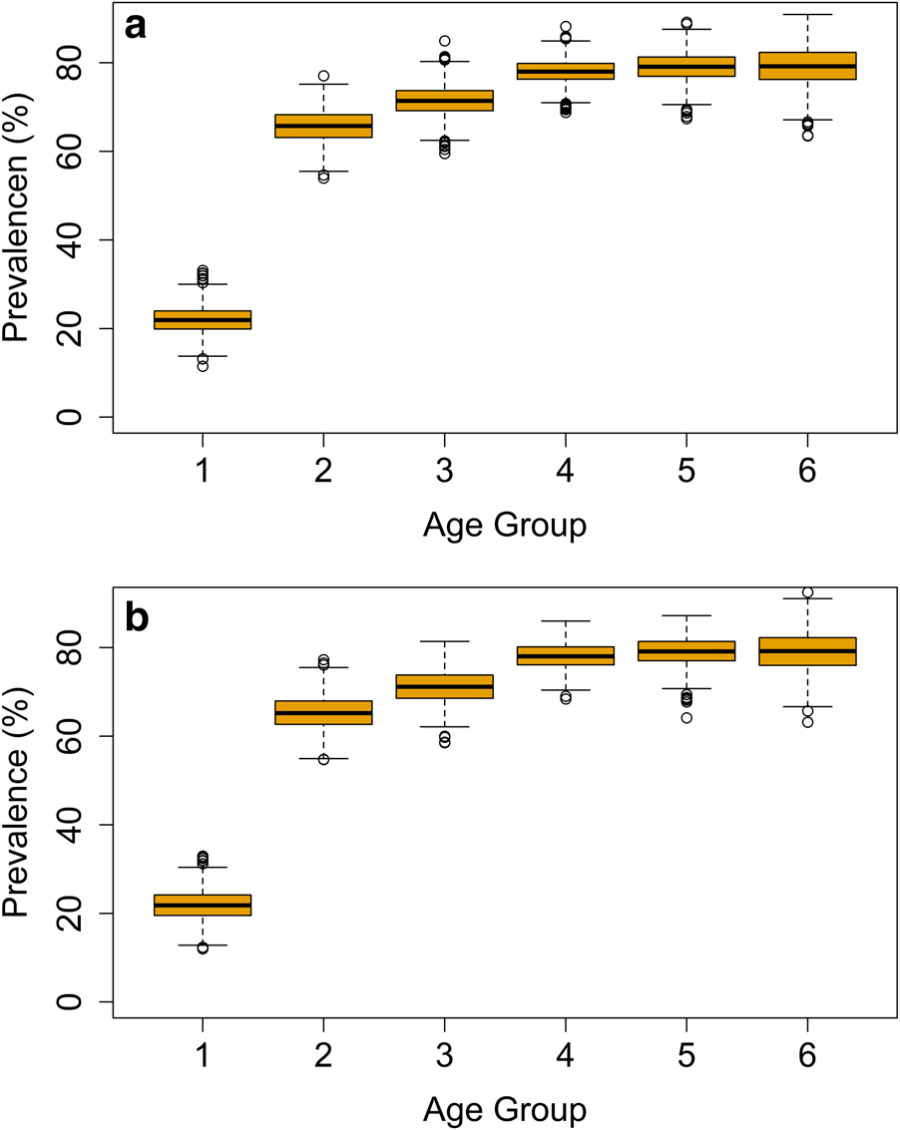
Age-prevalence over time in which elimination of *Necator americanus* infection was not achieved before mass drug administration was implemented (**a**) and post-MDA (**b**). Age groups (years): 1, 0–5; 2, 6–10; 3, 11–15; 4, 16–30; 5, 31–50; 6, 51+

The short- and long-term impact of treatment can also be observed in the age-prevalence surface plots (the dimensions of age and time). At equilibrium, the prevalence of hookworm infection was higher amongst adults than pre-SAC and SAC. Treatment resulted in a decrease in prevalence amongst all age groups, with the greatest relative decrease observed in adults. In villages which did not reach elimination and bounced-back to the pre-treatment equilibrium, the post-treatment age-prevalence profiles were near-identical to pre-treatment levels (Fig. 3, Additional file 1: Figure S2).

**Fig. 4:**
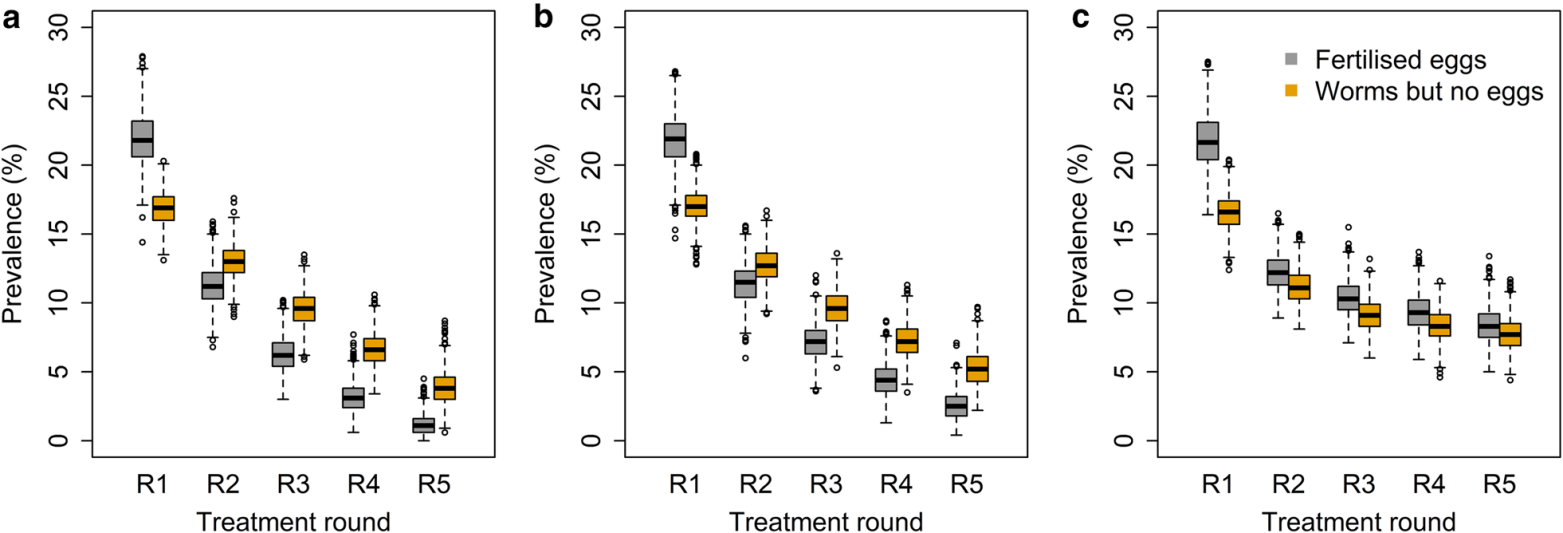
The proportion of individuals who harbour both male and female worms and produce fertile eggs (grey shaded color) and the proportion of individuals who harbour either only male or only females worms and produce no eggs (orange shaded color) for random compliance (**a**), semi-systematic compliance (**b**) and systematic compliance (**c**)

The proportion of individuals who still harbour worms after MDA showed differences between the compliance patterns types (Additional file 1: Figure S3). At round five, the proportion of individuals who harbour at least one worm were 5.1% (0.8–12.5%), 7.8% (2.9–16.6%) and 16.1% (10.4–24.1%) for random, semi-systematic and fully-systematic non-compliance (Additional file 1: Figure S3). The proportion of individuals who harbour both male and female worms reduced on average to 1.2% (0–4.5%) with random compliance (Fig. 4). With fully-systematic non-compliance, the proportion of individuals with both male and female worms remained high even after five rounds of MDA (8.4%, 5.0–13.4%) allowing transmission to be sustained. Similar results were found for *A. lumbricoides* (Additional file 1: Figures S3, S4).

### Simple theory compared with the outcomes predicted by the complex stochastic simulations

**Fig. 5:**
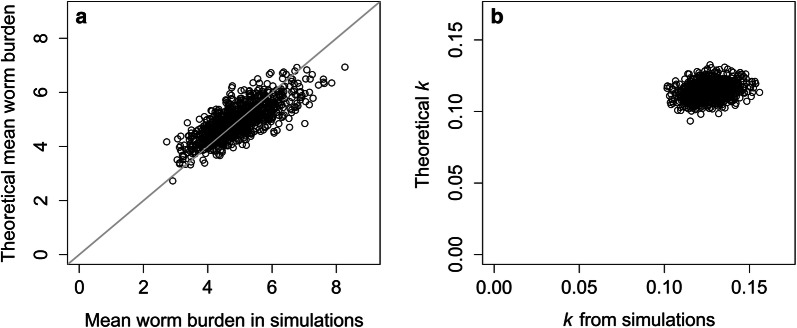
Results from analytical approach for predicting change in aggregation after treatment under semi-systematic non-compliance. **a** Comparison of mean worm burden observed from the stochastic model immediately post-treatment and that expected from the simple probability model. **b** Comparison of *k* observed from the stochastic model immediately post-treatment and that expected from the simple probability model. *Note*: for **a**, the grey line represents the equation x = y (perfect agreement)

The impact of MDA on the degree of aggregation was found to be described well by the simple probability model of compounding distributions created by heterogeneities within the host population due to either compliance to treatment or exposure to infection. For *N. americanus*, a high degree of agreement was seen between the mean worm burden and *k* values observed from the stochastic simulations immediately post-treatment and those expected under the simple probability model defined by the equations given in Additional file 2: Text S1. Strong agreement between observed and expected results was identified for *A. lumbricoides*, with the variance in worm count post-treatment showing a much stronger agreement between models than was found for *N. americanus* (Fig. 5; Additional file 1: Figure S5).

## Discussion

We have shown that the aggregation parameter *k* decreases immediately after each round of treatment reflecting increased parasite aggregation within the human host population. Furthermore, this decrease can be accurately predicted using a simple probability model which incorporates heterogeneities within human host populations created by either age-dependencies in worm burden (i.e. age-dependent exposure to infection) and/or variation in treatment coverage by age. In the long-term over multiple rounds of MDA, the decline in the value of *k* follows the decline in prevalence within any given village. Our results show that the *A. lumbricoides* simulations are more likely to achieve interruption of transmission for all three compliance types. This is likely to be the result of higher coverage in the SAC-population which have typically the highest *A. lumbricoides* worm burden. Whilst for hookworm the opposite is noticed. Moreover, the egg reduction rate is slightly higher for *A. lumbricoides* [40].

The simple model requires data describing the number of worms within each individual host both pre- and post-treatment. Worm expulsion post treatment is both time- and resource-intensive, by comparison with collecting data on egg counts *via* stool examination (typically through Kato-Katz, KK). Worm expulsion data can be unreliable since stool samples need to be collected for at least 6 consecutive days to collect at least 80% of *A. lumbricoides* worms post-treatment [41]. Given that hookworms are much smaller than *A. lumbricoides*, worm counts are considered even less precise as many worms are likely to be missed in the collected stool. Since worm expulsions are performed by giving individuals chemotherapeutic treatment, the collection of empirical “pre-treatment” worm counts is often not possible in current government run deworming treatment programmes. We can extract information on worm burden at any time point from the individual-based stochastic simulations models, but such data are difficult to obtain in ongoing field epidemiological studies. One potential solution (although by no means a perfect one) could be to compare post-treatment worm expulsion data with pre-treatment KK intensity data for the same individuals. Whilst this would not be “worm expulsions” at both time points and the two measures would not necessarily be directly comparable, this approach may be of help given good data on the relationship between worm burdens and egg counts.

Egg count data, collected at both pre- and post-treatment, can be used as a proxy for worm count data. However, microscopic diagnostic tools, such as KK, have a reduced sensitivity at low intensities of infections. Nikolay et al. [42] showed that whilst the sensitivity of double-slide KK (the most commonly used microscopic diagnostic tool) was acceptable for the three major STH species in high-intensity of infection areas (sensitivity of 74–95%), it was much less reliable in areas of low intensity (sensitivity of 53–80%), with the lowest values being for hookworm and *A. lumbricoides*. Therefore, these diagnostic tools are less suitable for populations that are close to reaching the breakpoint in transmission. The increasing use of MDA combined with the ongoing push for STH elimination will likely result in greater pressure on the use of more sensitive diagnostics to detect infection at lower prevalence and intensity. To this end, there has been increased interest in recent years in the potential use of quantitative polymerase chain reaction (qPCR) assays as a diagnostic tool for STH infections [43, 44]. This technique has greater sensitivity than KK especially when the prevalence and intensity of infection are both low [20, 43, 44]. The use of improved diagnostics with increased sensitivity will be very beneficial in areas approaching the breakpoint in transmission to ascertain if transmission has ceased. However, the increased costs of qPCR by comparison with KK may be a deterrent in low resource settings.

Calculating *k* values was found to be difficult for some villages in both the random and semi-systematic non-compliance simulations post-cessation of treatment due to very low average worm burdens such that very few individuals were infected. When all individuals within a village have either zero or one worm, as was the case in the villages for which *k* could not be estimated, the negative binomial distribution no longer describes the worm burden distribution and a Bernoulli distribution is a more appropriate probability model. In these circumstances, no negative binomial aggregation parameter could be derived and the maximum likelihood analysis was forced to select whichever value was set as the upper limit for *k* as the best fit (here, *k* = 0.5) in the estimation process. A fuller explanation is presented in Additional file 2: Text S1.

Aggregation of macroparasites within the human hosts is a commonly observed epidemiological feature. The degree of overdispersion can depend on the parasite species, host age and gender, and the transmission setting. For example, children are often predisposed to heavy *A. lumbricoides* infection. Predisposition to hookworm infection is also observed, but the factors that are linked to predisposition are not well understood at present [45]. It may be some combination of genetic, behavioural and environmental factors.

Understanding compliance within a study through the collection of individual-based, longitudinal treatment data is of the upmost importance. The probability of successfully eliminating *N. americanus* infection within our simulations with a fixed level of coverage was highly dependent on the pattern of compliance assumed, ranging from almost 90% with random compliance to 0% under systematic non-compliance. Therefore, it is crucial to the success of a deworming campaign aimed at breaking transmission that all individuals within a village are treated and that no individuals are systematically missed. This can occur when households are too remote, individuals persistently refuse treatment or if individuals are consistently not at home when health care workers are visiting. Knowledge of the factors that influence persistent non-compliance can help in the design and implementation of parasite control programmes.

Knowledge regarding the extent of compliance within a population targeted for treatment can contribute towards an understanding of whether or not to stop randomised STH control trials, such as DeWorm3, earlier than originally planned [18, 21]. If compliance is consistently poor there is little point in continuing treatment, monitoring and evaluation since transmission elimination is unlikely to occur.

Anthelmintic resistance is a significant problem in livestock farming and is increasingly reported amongst by the veterinary profession. It is important to note that drug pressure in livestock is much higher, in part as a consequence of livestock being treated five times a year and hence parasites have a high and repeated exposure to the drug selective pressure. Nonetheless, the risk of developing drug resistance in human STH is important to monitor when the goal is shifting from morbidity control towards interruption of transmission. This is especially true for species with a suboptimal response to the drugs, for example the response of *T. trichiura* to albendazole and mebendazole. With the shift towards interruption of transmission, drug pressure within a population increases substantially and concomitantly selection pressure on the parasite population. Even though there is widespread agreement regarding the importance of monitoring drug resistance, reliable studies investigating this challenge are very few at present. Microscopic diagnostic tools and qPCR alone are not sufficient to identify drug resistance, other methods such as population genetics are required. Worm DNA needs to be isolated from expulsion studies and the beta-tubulin gene (where resistance has been reported before) needs to be sequenced. One example of recent progress is the STARWORM project which aims to identify standardized methods to monitor drug resistance [46]. During the DeWorm3 trial drug resistance is being closely monitored and samples are being stored to investigate resistance mutations in future molecular epidemiological analyses. If drug resistance appears to develop in the DeWorm3 study sites, it will be important to understand why this is the case and monitoring changes in aggregation patterns can support the detection of drug resistance [18].

The effects of migration of infectious individuals on the likelihood of interrupting transmission are poorly understood at present for helminth control. However, much recent infectious disease research is focused on this issue employing simple or complex models of spatial structure and human and animal movement. In this study, we assumed that villages behave as independent units with no immigration or emigration of infectious individuals. Recent molecular epidemiological analyses performed by Easton et al. [47], showed that for *A. lumbricoides* infection in Bungoma (Kenya), the village is the most appropriate epidemiological unit for study, since molecular epidemiological analyses revealed that the vast majority of transmission takes place between village members. If there is a lot of migration between villages, the risk of transmission from one village to another increases. However, this risk will depend on a number of factors, including the prevalence of infection and its heterogeneity between villages, heterogeneity in treatment coverage and compliance between villages, the age-group that moves most between villages and also the age-intensity profiles. For example, if there is a high prevalence of *A. lumbricoides* in one village, there is a low risk of transmission between villages as a consequence of migration if adults are the major migratory group. Whilst for hookworm the opposite is true and reaching a breakpoint will be difficult if much adult movement takes place [48]. Coverage and compliance on the village-level or household-level are obviously of high importance. If for example, a few households refuse to take the treatments offered in MDA rounds, they may maintain a local reservoir of infection within a village, which can complicate reaching the transmission breakpoint. How serious this complication is, depends on the contact of other village members with non-compliant households. Data currently being collected during the DeWorm3 trials will be very beneficial in parameterizing spatially structured models and in refining programmes that aim to interrupt transmission of STH on large spatial scales.

In future research, it is important to adapt the methods outlined in this paper to allow for the use of egg counts (eggs per gram of faeces; EPG) as opposed to the worm counts (which require high resource allocation to ensure accuracy). Worm expulsion studies are considerably more time- and resource-intensive than obtaining faecal egg counts *via* Kato-Katz. Adapting the simple model, such that EPG data can be utilised, will allow for the methodology to be used more widely within STH studies.

## Conclusions

We have shown how the increased aggregation in STH infection post-treatment observed in empirical studies can be predicted using a simple probability model based on compounding distributions arising from heterogeneities due to age-dependent exposure to infection and/or different patterns of MDA uptake by individual people. Ongoing studies under the TUMIKIA and D3Worm3 projects will provide further information on patterns of individual compliance. For hookworm, infection individual compliance is thought to be the major cause of the observed patterns of increasing parasite aggregation with increasing numbers of MDA rounds in given communities. Increased aggregation of worms after many rounds of chemotherapy suggests that once the prevalence of infection has reached low levels an approach to target and treat those predisposed to infection and non-compliant to treatment may be highly beneficial.

## Supplementary information

**Additional file 1: Figure S1.***A. lumbricoides* prevalence over time (a–c) and aggregation (parameter k) over time (d–f), in this plot we show 500 out of 1000 simulated villages. a and d: Random non-compliance; b and e: Semi-systematic non-compliance; c and f: Fully systematic non-compliance. **Figure S2.** Age-prevalence over time for a selected village in which elimination of *A. lumbricoides* infection was not achieved before mass drug administration was implemented (a) and post-MDA and one in which elimination was not achieved (b). Age groups (years): 1, 0–5; 2, 6–10; 3, 11–15; 4, 16–30; 5, 31–50; 6, 51+. **Figure S3.** The proportion of individuals who still harbour worms, either hookworm (a) or *A. lumbricoides* (b) after each round of mass drug administration (T1–5) for random compliance, semi-systematic compliance and systematic compliance. **Figure S4.** The proportion of individuals who harbour both male and female worms (*A. lumbricoides*) and produce fertile eggs (grey shaded color) and the proportion of individuals who harbour either only male or only females worms and produce no fertile eggs (orange shaded color) for random compliance (a), semi-systematic compliance (b) and systematic compliance (c). **Figure S5.** Results from analytical approach for predicting change in aggregation after treatment under semi-systematic non-compliance. a Comparison of mean worm burden observed from the stochastic model immediately post-treatment and that expected from the simple probability model. b Comparison of k observed from the stochastic model and that expected from the simple probability model immediately post-treatment. *Note*: For a, the gray line represents the equation x = y (perfect agreement).

**Additional file 2: Text S1.** A simple analysis of the impact of treatment and heterogeneity on worm aggregation among hosts.

## Data Availability

Data supporting the conclusions of this article are included within the article and its additional files. Data from the simulation runs are available upon request from the corresponding author. All other data used in the analyses are included in the cited publications.

## Abbreviations

DALYs: disability-adjusted life years
EPG: eggs per gram of faeces
KK: Kato-Katz
MDA: mass drug administration
NTDs: neglected tropical diseases
SAC: school-aged children
STH: soil-transmitted helminths
qPCR: quantitative polymerase chain reaction
WHO: World Health Organisation
WRA: women of reproductive age
