# Supplementary material for: The impact of community-wide, mass drug administration on aggregation of soil-transmitted helminth infection in human host populations
Source: Parasit Vectors. 2020 Jun 8;13:290. doi: 10.1186/s13071-020-04149-4 (PMC7278197; doi:10.1186/s13071-020-04149-4)
Supplement: Supplementary file 1 — Additional file 1: Figure S1.A. lumbricoides prevalence over time (a–c) and aggregation (parameter k) over time (d–f), in this plot we show 500 out of 1000 simulated villages. a and d: Random non-compliance; b and e: Semi-systematic non-compliance; c and f: Fully systematic non-compliance. Figure S2. Age-prevalence over time for a selected village in which elimination of A. lumbricoides infection was not achieved before mass drug administration was implemented (a) and post-MDA and one in which elimination was not achieved (b). Age groups (years): 1, 0–5; 2, 6–10; 3, 11–15; 4, 16–30; 5, 31–50; 6, 51+. Figure S3. The proportion of individuals who still harbour worms, either hookworm (a) or A. lumbricoides (b) after each round of mass drug administration (T1–5) for random compliance, semi-systematic compliance and systematic compliance. Figure S4. The proportion of individuals who harbour both male and female worms (A. lumbricoides) and produce fertile eggs (grey shaded color) and the proportion of individuals who harbour either only male or only females worms and produce no fertile eggs (orange shaded color) for random compliance (a), semi-systematic compliance (b) and systematic compliance (c). Figure S5. Results from analytical approach for predicting change in aggregation after treatment under semi-systematic non-compliance. a Comparison of mean worm burden observed from the stochastic model immediately post-treatment and that expected from the simple probability model. b Comparison of k observed from the stochastic model and that expected from the simple probability model immediately post-treatment. Note: For a, the gray line represents the equation x = y (perfect agreement). [file 13071_2020_4149_MOESM1_ESM.docx]

# **Additional file 1**

## ***Ascaris:* Observations from stochastic simulations**

At equilibrium, prevalence was a little over 30% in all villages for all compliance scenarios. After the cessation of treatment, village prevalence either recovered to equilibrium levels or decreased to zero, depending on whether the breakpoint in transmission was crossed (= the unstable equilibrium). The proportion of villages achieving elimination was 75.6% under random compliance, 65.7% for semi-systematic, and 43.2% for systematic compliance **[Figures S1a-c].**

Under random compliance, $k$ decreased sharply to between 0.048 and 0.099 directly following each round of treatment, before returning to near-equilibrium levels almost immediately. Similar patters were also observed in both the systematic (range: 0.048- 0.092) and semi-systematic (range: 0.051-0.092) non-compliance scenarios. In all cases, the range in *k* values increased slightly after each successive round of treatment. In villages where elimination of transmission was successful (the “breakpoint” in transmission was crossed), and prevalence declined towards zero, $k$ tended to zero. In those villages which failed to reach elimination and suffered bounce-back in infection to pre-treatment equilibrium levels, $k$ was observed to do the same. No significant difference between compliance schema with regards the duration of time required for $k$ to return to pre-treatment equilibrium levels was observed **[Figures S1d-f]**.


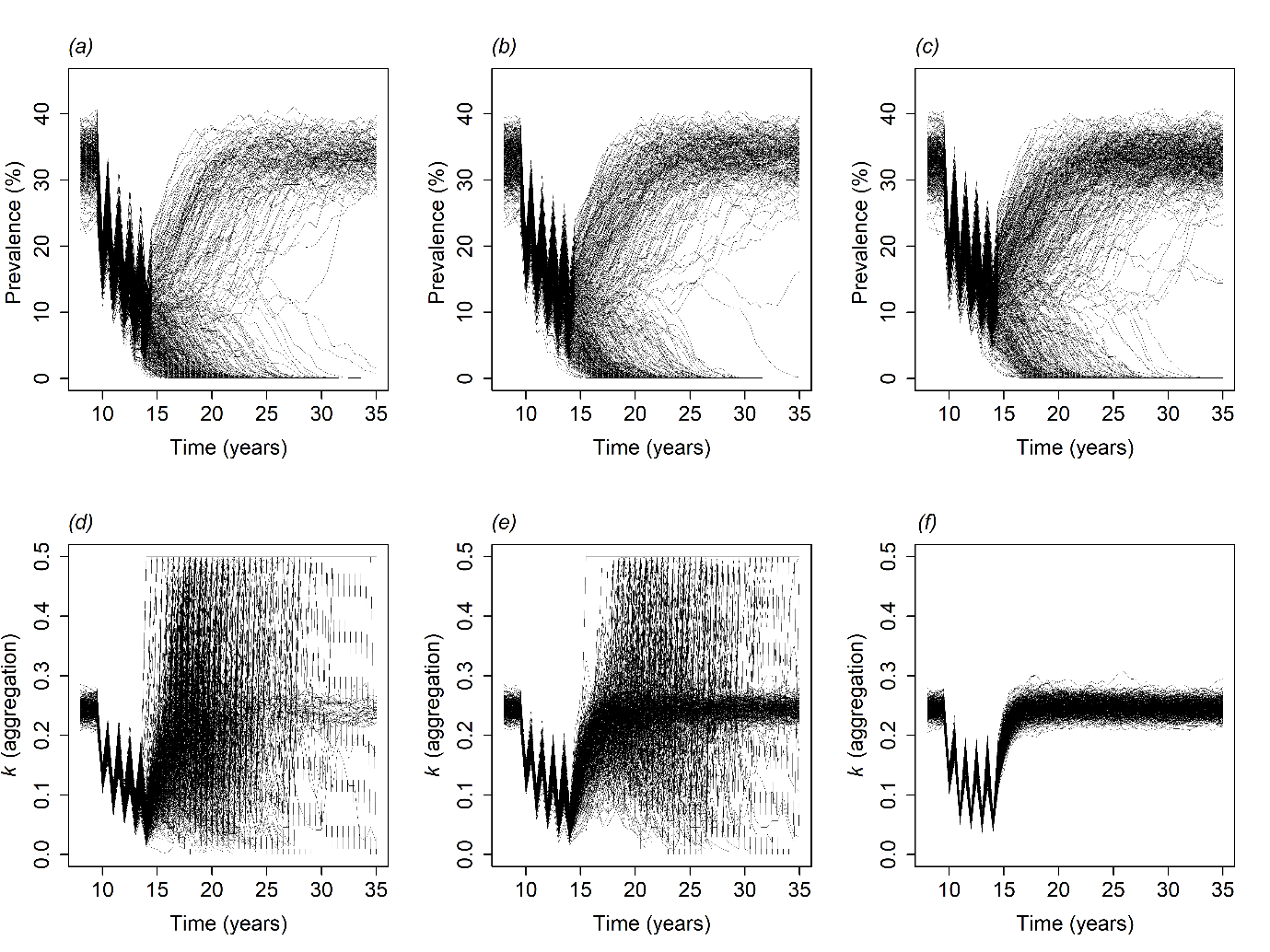


**Figure S1. A. lumbricoides prevalence over time (a-c) and aggregation (parameter k) over time (d-f), in this plot we show 500 out of 1,000 simulated villages. a and d: Random non-compliance; b and e: Semi-systematic non-compliance; c and f: Fully systematic non-compliance.**

The short- and long-term impact of treatment can also be observed in the age-prevalence surface plots. At equilibrium, prevalence was generally higher in pre-SAC and SAC than in adults. Treatment resulted in a decrease in prevalence amongst all age groups, with the greatest decrease observed in pre-SAC and SAC, in line with the coverage levels implemented in the models. In villages which did not reach elimination, and witnessed bounce-back to equilibrium prevalence levels, the expected age-prevalence profiles were observed, with prevalence amongst pre-SAC and SAC being higher than that for adults **[Figures S2].**


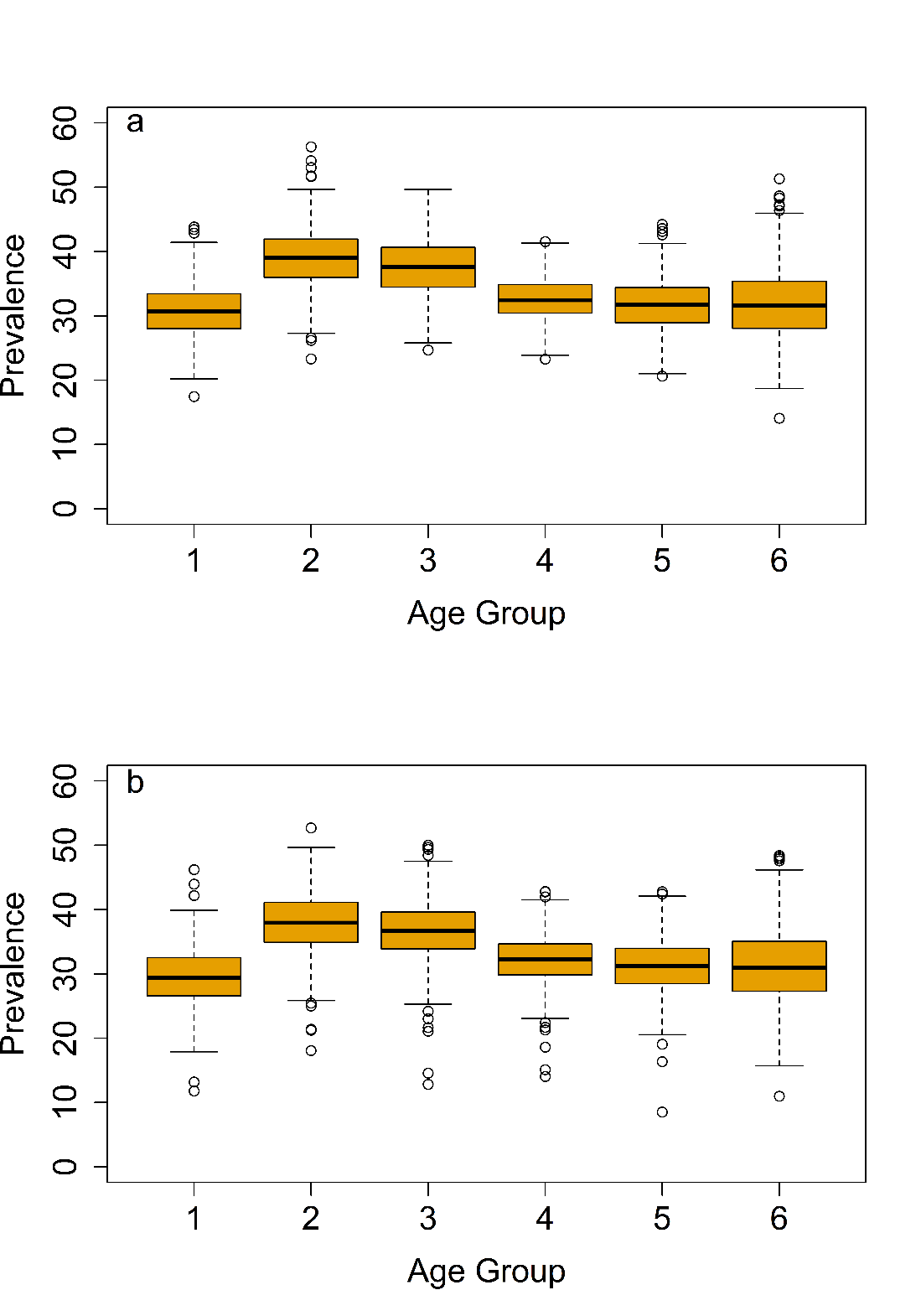


***Figure S2. Age-prevalence over time for a selected village in which elimination of A. lumbricoides infection was not achieved before mass drug administration was implemented (a) and post-MDA and one in which elimination was not achieved (b). Age groups (years): “1”: 0-5; “2”: 6-10; “3”: 11-15; “4”: 16-30; “5”: 31-50; “6”: 51+.***

**
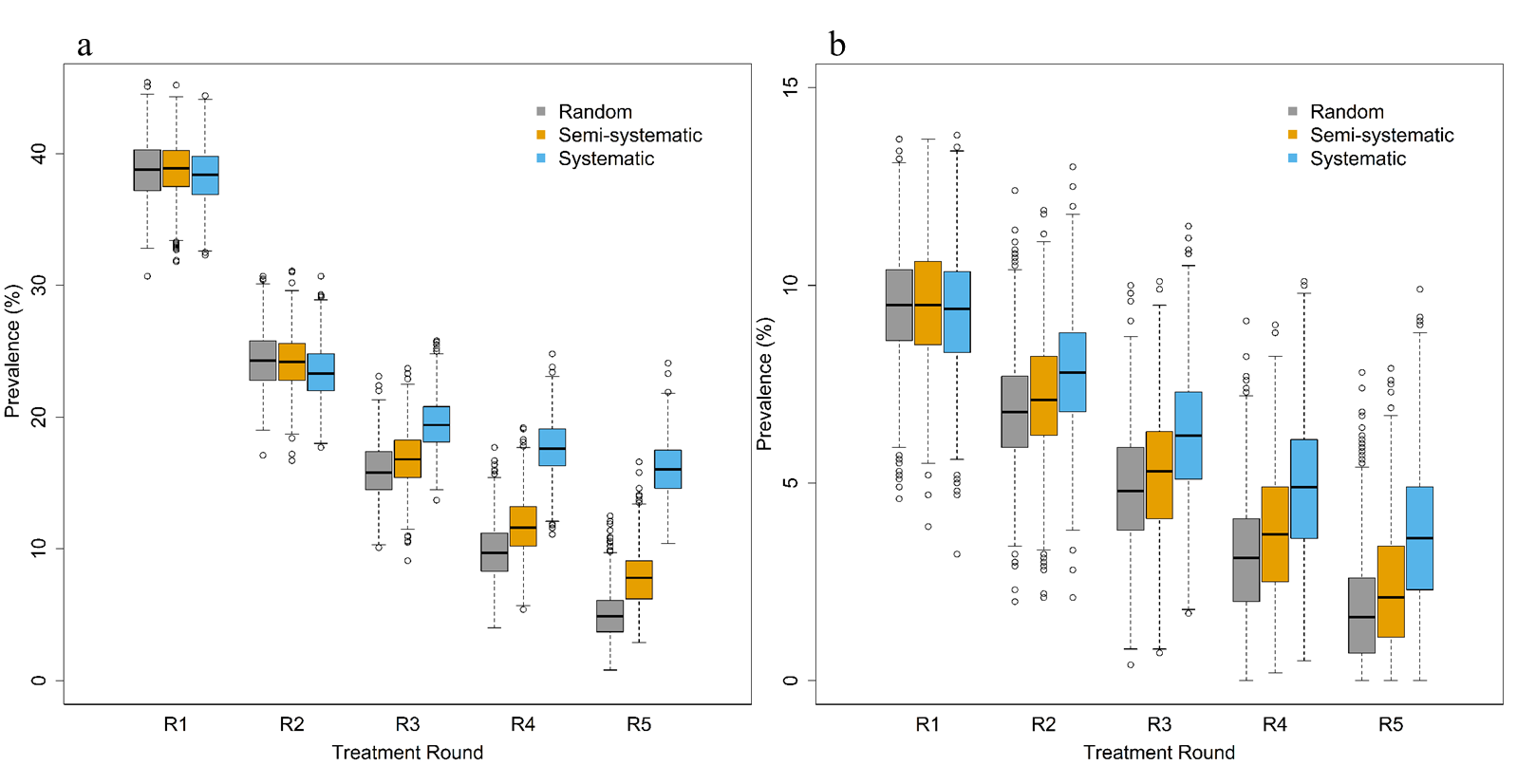
**

***Figure S3.*** ***The proportion of individuals who still harbour worms, either hookworm (a) or A. lumbricoides (b) after each round of mass drug administration (T1-5) for random compliance, semi-systematic compliance and systematic compliance.***


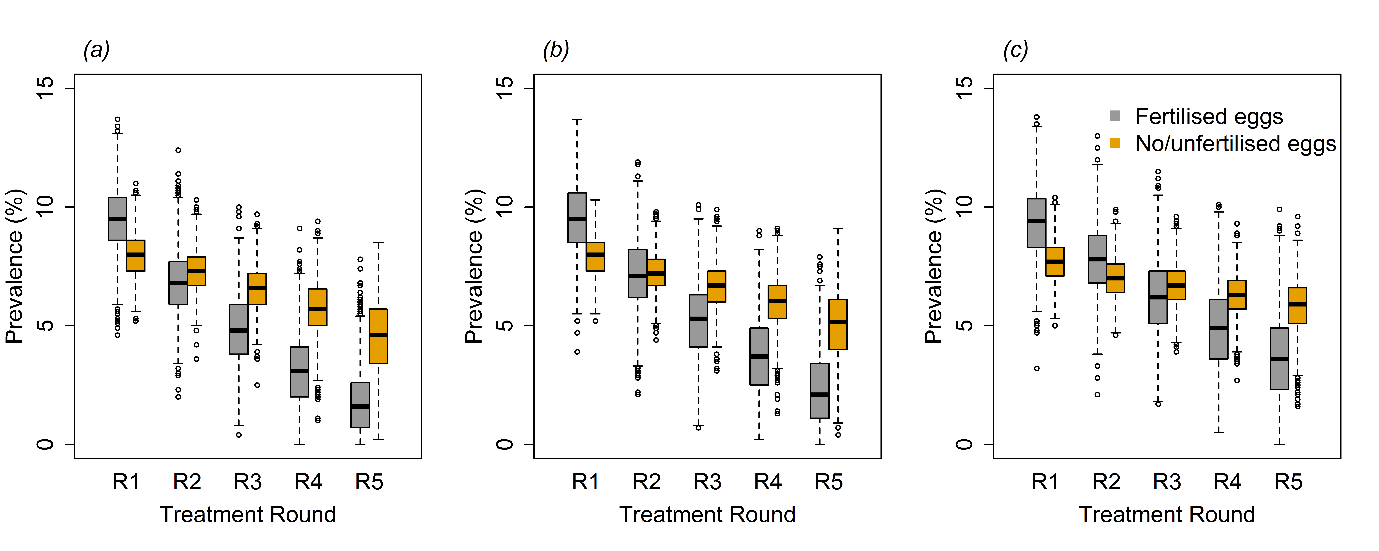


**Figure S4. The proportion of individuals who harbour both male and female worms (*A. lumbricoides*) and produce fertile eggs (grey shaded color) and the proportion of individuals who harbour either only male or only females worms and produce no fertile eggs (orange shaded color) for random compliance (a), semi-systematic compliance (b) and systematic compliance (c).**

## **Theory applied to stochastic simulations**

The impact of MDA on the degree of aggregation was found to be described well by a simple probability model (see S1). A strong association was seen between the mean worm burden, variance in worm numbers, and $k$ values observed from the stochastic simulations immediately post-treatment and those expected under the equations defined in the SI material [Figure S2.6].


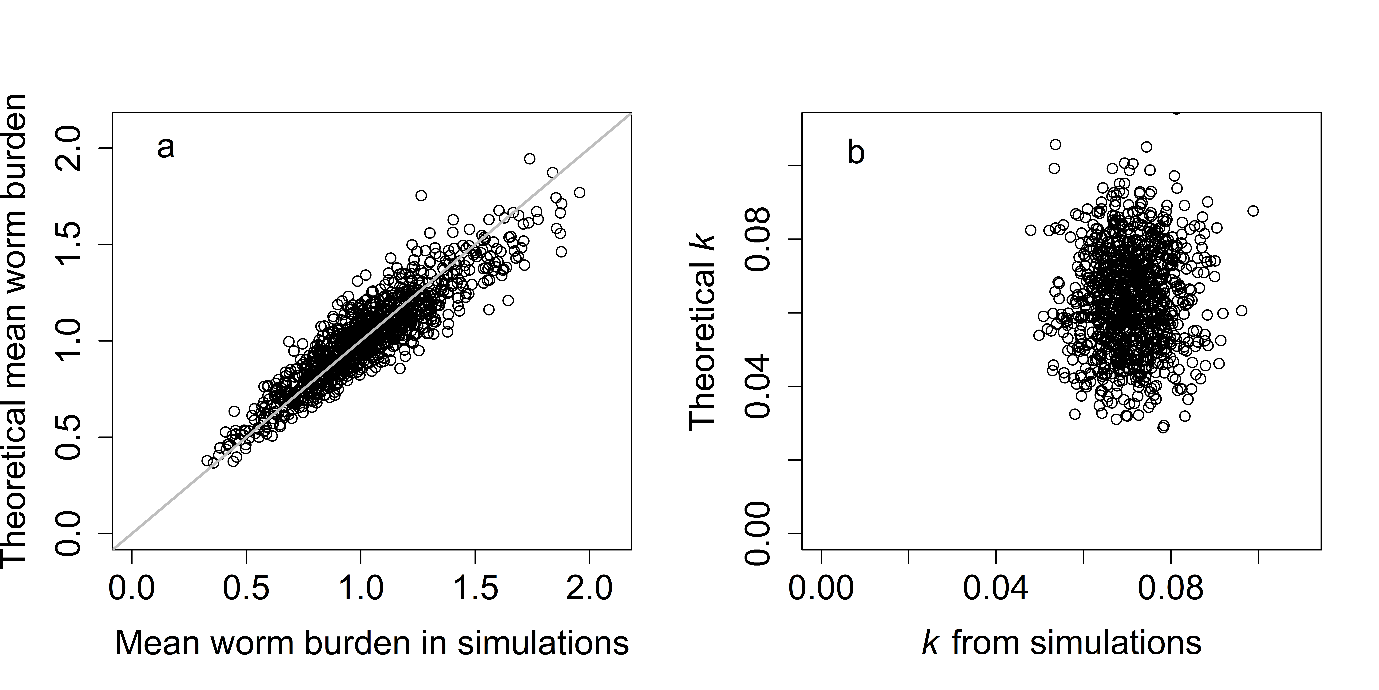


**Figure S5. Results from analytical approach for predicting change in aggregation after treatment under semi-systematic non-compliance. a Comparison of mean worm burden observed from the stochastic model immediately post-treatment and that expected from the simple probability model. b Comparison of k observed from the stochastic model and that expected from the simple probability model immediately post-treatment. NB: For a, the gray line represents the equation x = y (perfect agreement).**
