# Supplementary material for: The impact of community-wide, mass drug administration on aggregation of soil-transmitted helminth infection in human host populations
Source: Parasit Vectors. 2020 Jun 8;13:290. doi: 10.1186/s13071-020-04149-4 (PMC7278197; doi:10.1186/s13071-020-04149-4)
Supplement: Supplementary file 2 — Additional file 2: Text S1. A simple analysis of the impact of treatment and heterogeneity on worm aggregation among hosts. [file 13071_2020_4149_MOESM2_ESM.docx]

# **Additional file 2: Text S1**

# **A simple analysis of the impact of treatment and heterogeneity on worm aggregation among hosts**

## **Background**

Here we outline a simple probability model of the change in worm aggregation among hosts that results from heterogeneity in exposure to infection or the impact of drug treatment. Within a defined host grouping (in terms of age or treatment compliance and coverage), the parasite burden among hosts within the model is assumed to be precisely negative binomial (NB) in form. If we consider the overall distribution of worms across hosts that have different mean burdens (due to age-dependent exposure) or across populations that have imperfect treatment coverage, the new distribution will comprise of a set of negative binomial distribution with the overall distribution resulting from compounding the different negative binomial distributions (with different means and variances) in each exposure to infection or treatment compliance or coverage category. The resultant distribution is again negative binomial in from. We can calculate the mean and variance of the new compound distribution and using the negative binomial relationship between mean, variance and k,

$$\sigma^{2}=\frac{\mu(\mu+k)}{k}$$

to calculate the effect aggregation parameter for the new compound distribution. That is,

$k\simeq\frac{\mu^{2}}{\sigma^{2}-\mu}.$

## **The impact of treatment on measured parasite aggregation**

Focussing on the impact of treatment, we assume that in sufficiently small host age ranges, the mean worm burden is constant, giving a negative binomial worm distribution within that age group. We further assume that treatment kills worms with a probability given by the drug efficacy, e_f_, independent of the number of worms present. As a result, if the population of worms in a population is NB with parameters (*m*,*k*) before treatment, then after treatment it will still have a NB distribution, but with parameters ((1-e_f_)*m*,*k*). Post treatment, we can then calculate the mean and variance of the worm burden in the combined treated and untreated population and then use the expression in (1.1) to estimate the apparent value of the new aggregation parameter for the treated and untreated population.

Consider a group of individuals subject to a round of chemotherapy with efficacy, e_f_ and coverage *c*. Individuals sampled from this group will have a probability *c* of receiving the drug. In treated individuals, worms have a probability e_f_ of being killed. Given a pre-treatment mean burden of m_0_, after treatment, a host has a probability *c* of being in a group with mean burden e_f_m_0_ and a probability 1-*c* of retaining a mean burden of m_0_. Hence, sampling from a population subject to this treatment regime, the mean worm burden for an individual, *m*, is

$$m\sim m_{0}(1-e_{f}Bern(c))$$

where *Bern* is the Bernoulli distribution. So

$$\bar{m}=m_{0}(1-e_{f}c)$$

$$\sigma_{m}^{2}=m_{0}^{2}e_{f}^{2}c(1-c).$$

Prior to treatment, *m* = m_0_ and is not a random variable, therefore its variance is zero.

For a compound random variable, *x(y)*, where *y* is also a random variable, the variance is given by

$$Var[x]=E_{y}[Var_{x}[x(y)]]+Var_{y}[E_{x}[x(y)]]$$

In this case, the number of worms, $w\sim\text{NB}(m,k)$ and the distribution of *m* is given by equation 1.2. So

$Var[w] = E_{m}\left[ Var_{w}\left[ w\left( m \right) \right] \right] +\text{ Va}\text{r}_{m}[E_{w}\left[ w\left( m \right) \right]]$

$Var[w] = E_{m}\left[ Var_{w}\left[ w\left( m \right) \right] \right] +\text{ Va}\text{r}_{m}[E_{w}\left[ w\left( m \right) \right]]$

$$=\text{ }E_{m}\left[m\left(m+k\right)/k\right]\text{ }+\text{ Var}_{m}[m]$$

$$=\left(1+\frac{1}{k}\right)\sigma_{m}^{2}+\frac{1}{k}\bar{m}^{2}+\bar{m}.$$

Substituting in the values for the distribution of *m* gives

$$Var[w]=\left(1+\frac{1}{k}\right)m_{0}^{2}e_{f}^{2}c(1-c)+\frac{1}{k}m_{0}^{2}(1-e_{f}c)^{2}+m_{0}(1-e_{f}c)$$

The mean of *w* is $\bar{w}=\bar{m}=m_{0}(1-e_{f}c)$. Using the relationship in equation 1.1,

$$k'\simeq k\frac{(1-e_{f}c)^{2}}{\left(k+1\right)e_{f}^{2}c(1-c)+(1-e_{f}c)^{2}}$$

This represents the change in apparent aggregation across one round of treatment. Aggregation therefore always increases (*k* decreases) immediately post treatment. Note that the change in aggregation is independent of the initial mean worm burden and for *k* << 1, it is also independent of the degree of initial aggregation prior to treatment.

## **Is overall worm aggregation a function of force of infection?**

It has been observed that the aggregation of worms amongst hosts appears to increase as prevalence decreases [1, 2]. It is informative to ask if the STH transmission model as currently formulated will reproduce this phenomenon. Within the model, the force of infection experienced by a host of a given age is given by

$$\lambda(a)=q\beta(a)$$

where $\beta(a)$is the age-dependent contact rate and *q* is the intensity of infectious material in the environment. The mean worm burden at age a is given by

$$m(a)=\int_{s=0}^{\infty} \lambda(a-s)e^{-\sigma s}ds\propto q$$

assuming that $\lambda(a)=0,a<0$. So mean worm burden at a given age is proportional to intensity of infectious material. The prevalence in a population with a given age-dependent contact profile is a non-linear function of the mean worm burden with age and hence of intensity of infectious material in the environment, q, as well.

$$\pi=\int_{s=0}^{\infty} p(a)[1-(1+m(a)/k)^{-k}]da$$

Using the variance expression in equation 1.3, worm burden variance across all age groups is given by

$$Var[w]=\left(1+\frac{1}{k}\right)Var[m]+\frac{1}{k}\bar{m}^{2}+\bar{m}$$

The denominator of the expression for estimating k,$\sigma^{2}-\mu$ (equation 1.1), and the numerator are both proportional to *q^2^*. As a result, the estimated aggregation is independent of the changing force of infection and hence also independent of the overall prevalence. If aggregation is to change as prevalence decreases, the relative force of infection between different age groups (or other subgroups in the population) must also change. This is an indication that an additional, small-scale, level of heterogeneity is present in the host population with respect to force of infection, possibly focussed on groupings like households or schools.

# **Estimating aggregation at low prevalence**

Estimation of the aggregation parameter, *k*, from simulation data is based on the negative binomial likelihood function. As worm populations reach very low prevalences, estimation of *k* becomes impossible. A brief examination of the likelihood function shows why. Consider data from a population of *N* hosts, in which the prevalence is so low that individuals have only one or zero worms. If there are $n_{+}$ individuals with a single worm, the prevalence will be $\mu=n_{+}/N$. The log-likelihood of the data is

The maximum likelihood estimator for $ k $ is given by the derivative of the likelihood

It is easy to show that the derivative is always positive, except for a single point of infection at $k=\mu+k$. As a result, there is no local minimum and hence no maximum likelihood estimator for *k*. The likelihood continues to increase with increasing *k*. Attempts to estimate *k* using likelihood methods at very low prevalences are very likely to result in estimates for *k* that rise asymptotically. The appearance of this rise indicates that there is insufficient information in the data to estimate the aggregation pattern of the distribution.

# References

1. Guyatt HL, Bundy DAP, Medley GF, Grenfell BT. The relationship between the frequency distribution of Ascaris lumbricoides and the prevalence and intensity of infection in human communities. Parasitology. 1990;101 Pt 1:139–43. http://www.ncbi.nlm.nih.gov/pubmed/2235069. Accessed 26 Nov 2013.

2. Lwambo NJ, Bundy DAP, Medley GF. A new approach to morbidity risk assessment in hookworm endemic communities. Epidemiol Infect. 1992;108:469–81. http://www.pubmedcentral.nih.gov/articlerender.fcgi?artid=2272209&tool=pmcentrez&rendertype=abstract. Accessed 23 Jun 2014.
